# The expression of miR-17 and miR-29a in placenta-derived exosomes in LPS-induced abortion mice model: An experimental study

**DOI:** 10.18502/ijrm.v19i5.9252

**Published:** 2021-06-23

**Authors:** Tahereh Jalilvand, Reza Salarinia, Hasan Namdar Ahmadabad, Mohammadreza Safdari

**Affiliations:** ^1^Student Research Committee, School of Medicine, North Khorasan University of Medical Sciences, Bojnurd, Iran.; ^2^Department of Advanced Sciences and Technologies, School of Medicine, North Khorasan University of Sciences, Bojnurd, Iran.; ^3^Department of Pathobiology and Laboratory Sciences, School of Medicine, North Khorasan University of Medical Sciences, Bojnurd, Iran.; ^4^Department of Orthopedic Surgery, Immam Ali Hospital, North Khorasan University of Medical Sciences, Bojnurd, Iran.

**Keywords:** Exosome, miR-17, miR-29a, Placenta, Inflammation.

## Abstract

**Background:**

The expression pattern of microRNAs in placenta-derived exosomes plays a crucial role in the regulation of immune responses and inflammation at the fetal–maternal interface.

**Objective:**

Considering the immunomodulatory properties of miR-17 and miR-29a, we determined their expression levels in placenta-derived exosomes in a lipopolysaccharide (LPS)-induced abortion mice model.

**Materials and Methods:**

A total of 14 pregnant BALB/c mice, aged 6–8 wk, were randomly divided into two groups (n = 7/each) on the gestational day 11.5. While the mice in the experimental group were treated with LPS, those in the control group were treated with Phosphate buffered saline; 5 hr after the treatment, the placental cells were isolated and cultured for 48 hr. Then, the cell culture supernatants were collected and used for isolation of exosomes. The isolated exosomes were confirmed by western blot and scanning electron microscopy. The miRNAs were then extracted from exosomes, and cDNA synthesized. The expression levels of miR-17 and miR-29a were evaluated by quantitative real-time PCR analysis.

**Results:**

Our results showed that the expression levels of miR-29a in placenta-derived exosomes obtained from the experimental group increased significantly compared to the control group. Also, the expression levels of miR-17 in the placenta-derived exosomes obtained from the experimental group were found to decrease; however, it did not show significant changes compared with the control group (p > 0.05).

**Conclusion:**

Inflammatory reactions at the fetal–maternal interface can alter miRNAs expression patterns in placenta-derived exosomes, especially miRNAs with immunomodulatory effects such as miR-29a.

## 1. Introduction

Pregnancy is an individual condition of immunomodulation in which maternal immune responses adapt to the semi-allogenic fetus and preserve its efficiency to fight against the external pathogens (1). This has been achieved by massive immune cells infiltration into the fetal–maternal interface and also soluble factors secreted by them (2, 3). During pregnancy, placenta cells regulate maternal immune responses by releasing various soluble factors such as hormones, chemokines, cytokines, and extracellular vesicles. Exosomes are a kind of extracellular vesicles secreted by placenta cells under both physiological and pathological conditions (4). These are 40–100 nm in size and have a complex composition of proteins, lipids, and nucleic acids, including mRNAs, small fragments of DNA, and non-coding RNAs (most importantly miRNAs) (5). Exosome-derived miRNAs have an immunoregulatory effect on the immune cells through target mRNAs that encode proteins involved in Toll-like receptor (TLR) signaling, chemokine/cytokine signaling, and NFκB signaling pathway (6). It is well-established that changes due to infection, inflammation, or transformation can influence and thereby alter exosome-derived miRNAs (7). Maternal genital tract infections caused by gram-negative bacteria frequently occur during pregnancy and associate with a variety of pregnancy complications, such as fetal growth restriction, preterm labor, abortion, and preeclampsia (8, 9). Lipopolysaccharide (LPS) is an important cell-wall component of gram-negative bacteria that induces proinflammatory responses by engagement of TLR-4 (10). Considering that TLR4 is expressed in placenta cells, exposure to LPS of gram-negative bacteria can stimulate signaling pathways in them that activate various genes involved in inflammatory reactions (11). We assumed that exposure to LPS during pregnancy could alter the composition of placenta-derived exosomes, especially patterns of their miRNAs. Because of the presence of miRNAs encoded by miR-17-92 and miR-29 clusters in placenta-derived exosomes and their known immunomodulatory activities at the fetal–maternal interface (12, 13), we aimed to evaluate the expression levels of miR-17, a member of the miR-17-92 cluster, and miR-29a, a member of the miR-29 cluster, in placenta-derived exosomes in an LPS-induced abortion mice model.

## 2. Material and Methods

### Mice and mating

This experimental study was performed between 2017 and 2019 at the Department of Pathobiology and Laboratory Sciences in North Khorasan University of Medical Sciences, Bojnourd, Iran. A total of 14 female BALB/c mice, aged 6–8 wk were obtained from Razi Institute (Mashhad, Iran). All mice were maintained under a 12 hr light/dark cycle, with ad libitum access to food and water at 22–24°C with 40–60% humidity. For determining the gestational age, virgin female BALB/c mice were mated with male BALB/c, and the presence of a copulatory plug in the vagina was considered as day 0.5 of pregnancy.

### Animal treatment and sample collection

On gestation day 11.5, pregnant mice were randomly divided into two groups (n = 7/each). The mice in the experimental group were intraperitoneally injected with 2 µg/g (0.1 ml) LPS (from *Escherichia coli* O111: B4, Sigma, St. Louis, MO) (9), whereas the mice in the control group were intraperitoneally injected with an equivalent amount of Phosphate buffered saline (PBS). Next, 5 hr after the injection, pregnant mice from both groups were sacrificed, and their uteri were collected. We incised the uterus and collected placenta tissues as described by Mahdavi Siuki et al. (9).

### Extraction of exosomes from placenta cell culture

The placenta cell supernatant preparation was conducted as previously explained (14). In addition, exosomes were extracted from placenta cell supernatant according to a method described by Zeringer and coworkers (15).

### Exosome confirmation analysis 

Exosome morphology was confirmed by electron microscopy, and exosome-specific proteins were analyzed by western blot.

### Electron microscopy

After fixation of a portion of the purified exosomes using 2.5% glutaraldehyde, we dehydrated samples with mounting grades of ethanol. Samples were then vacuum-dried on a glass surface, and sputter-coated with gold. To confirm the size and shape of the exosomes, scanning electron microscopy was used (Hitachi S-4800, Tokyo, Japan). These methods have been described in more detail by Yazarlou and colleagues (16).

### Western blot analysis

To confirm the success of exosome isolation, we used western blot analysis. For this purpose, we lysed isolated exosomes in SDS-PAGE sample buffer (reducing) and boiled it at 95°C for 10 min. Then, proteins that resolved using SDS-polyacrylamide gel electrophoresis were transferred to nitrocellulose membranes (45 µm, Thermoscience, USA). The membranes were blocked with 5% skim milk in PBS with Tween 20 for 1 hr. After incubation, nitrocellulose membranes with target of the antiproliferative antibody 1 (TAPA-1) (Abcam, USA) for 1 hr, we incubated them with secondary HRP-conjugated antibody (Abcam, USA). Finally, the corresponding immunoreactive bands were developed using an electrochemiluminescence (ECL). The molecular weights of proteins were determined using the pre-stained protein ladder (SinaClon, Iran). This method is similar to that used by Lobb et al. (17).

### Exosomal RNA and microRNA extraction 

The miRNA was isolated from exosomes using the GeneAll Hybrid-R${}^{\mathrm{TM}}$ miRNA extraction kit (GeneAll, Republic of Korea) for small RNAs purification according to the manufacturer's instruction.

### RT-PCR reaction and cDNA synthesis

For mRNAs and microRNAs reverse transcription, isolated RNAs were reverse-transcribed using specific reverse transcriptase (RT) stem-loop primers for miR-17, miR-29a, and U6 small nuclear RNA (RNU6) (Table I) through HyperScriptTM First-strand Synthesis Kit (GeneAll, Republic of Korea) according to the manufacturer's guidelines.

The cDNA synthesis reactions included 3 µl RNA, 1 µl stem loop primers, 1 ml deoxynucleotide triphosphate (dNTPs) (10 mM), 9 µl nuclease-free water mixed, and incubated at 65°C for 5 min and kept on ice. Then, 2 µl RT reaction buffer (10x), 2 µl dithiothreitol (DTT) (0.1M), 1 µl moloney murine leukemia virus (MMLV) of reverse transcriptase (200 U/µl), and 1 µl ZymAll${}^{\mathrm{TM}}$ RNase Inhibitor were added to the reaction. The total mentioned agents (20 µl) were incubated for 15 min at 25°C, 15 min at 37°C, and 45 min at 42°C. The reaction was terminated by incubating at 85°C for 5 min and kept at –70°C until use.

### Quantitative real-time PCR analysis

We evaluated the expression levels of target microRNAs using quantitative real-time PCR. Each 25 µl PCR mixture contained 12.5 µl of PCR master mix (Ampliqon, Denmark), approximately 2 μL of synthesized cDNA (20 ng), 0.5 μL each of primer (0.1 μM), and 9.5 µl RNase-free water.

All experiments were run at least in triplicates. The amplification and detection of specific products were performed on the Rotor-Gene 6000 real-time PCR system (QIAGEN, USA). After an initial denaturation step at 95°C for 15 min, we used a denaturation step at for 30 sec, an annealing step at 60°C for 30 sec, and an elongation step at 72°C for 30 sec, for a total of 50 cycles. The relative expression of each miRNA was normalized using the cycle threshold (Ct) obtained from RNU6 as a housekeeping control gene and measured using the ΔΔCT method. The primer sequences are listed in Table I.

**Table 1:** The sequences of primers of miRNAs


**miRNA**	**Reaction**	**Primer sequence (5'-3')**
**miR-17**	RT	GTCGTATCCAGTGCAGGGTCCGAGGTATTCGCACTGGATACGACCTACCT
**miR-29a**	RT	GTCGTATCCAGTGCAGGGTCCGAGGTATTCGCACTGGATACGACTAACCG
**RNU6**	RT	CGAATTTGCGTGTCATCCTTGCG
**miR-17**	qPCR	Forward: CCGCCAAAGTGCTTACAGTGC Reverse: TATCCAGTGCAGGGTCCGAG
**miR-29a**	qPCR	Forward: CGCCTAGCACCATCTGAAATCG Reverse: TATCCAGTGCAGGGTCCGAG
**RNU6**	qPCR	Forward: CGGCAGCACATATACTAAAATTGGAACG Reverse: CGAATTTGCGTGTCATCCTTGCG
RNU6: U6 small nuclear RNA, RT: Reverse transcriptase, qPCR: Quantitative polymerase chain reaction		

### Ethical considerations

All experimental protocols used in this research were approved by the Ethics Committee of North Khorasan University of Medical Sciences, Bojnord, Iran (IR.nkums.REC.1395.100).

### Statistical analysis

Data were analyzed using IBM SPSS software, version 20 (SPSS, Armonk, NY, USA). Data were representative of two independent experiments and presented as means ± SEM. The differences between the two groups were examined using the student *t* test. P < 0.05 was considered as statistically significant.

## 3. Results 

**Figure 1:**
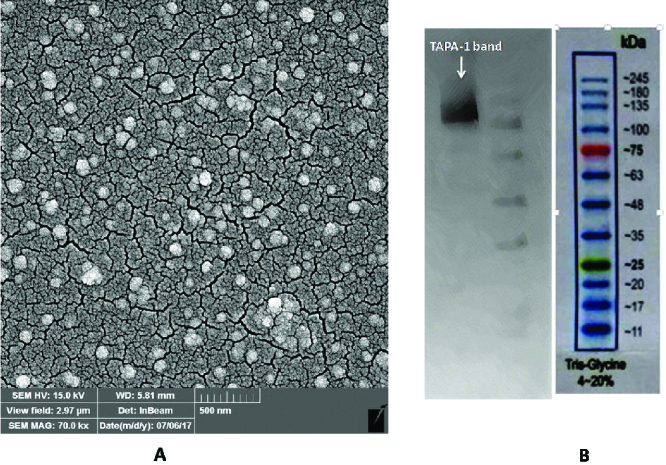
Confirmation of exosome isolation through scanning electron microscopy and western blot analyses. (A) The purified exosomes were fixed, dehydrated, and dried on a glass surface and sputter-coated with gold. The size and shape of purified exosomes were evaluated using SEM at (at scale = 500 nm, magnification = 70000×). (B) The presence of TAPA-1 as exosome-specific surface marker was analyzed through western blot.

After the extraction of exosomes from placenta cells supernatant, their size and shape were confirmed through scanning electron microscopy and the expression of TAPA-1 as exosome-specific surface marker through western blot (Figure 1).

**Figure 2:**
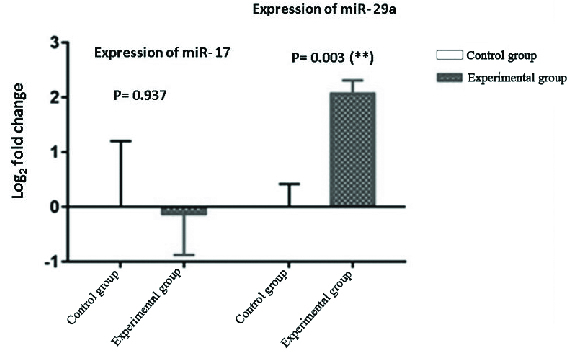
The effect of LPS treatment on the expression levels of miR-29a and miR-17 in BALB/c pregnant mice. Data are presented as Mean ± SEM (n = 7). Results are representative of three independent times for each experiments. **P < 0.01 was considered as statistically significant.

The expression levels of miR-29a and miR-17 in placenta-derived exosomes were determined by quantitative real-time PCR. Our results showed that LPS significantly increase the expression of miRNA-29a (4.16-fold) in placenta cells isolated from LPS-treated pregnant mice in experimental

group compared with the control group. We also found that the expression levels of miR-17 decrease in placenta-derived exosomes obtained from LPS-treated pregnant mice in experimental group, but we did not observe a significant difference compared with the PBS-treated pregnant mice in control group (Figure 2).

## 4. Discussion

The results of this study showed that treating pregnant BALB/c mice with LPS decreased the expression levels of miR-17 in placenta-derived exosomes, but this difference was not statistically significant. We also found that this treatment significantly increases the expression levels of miRNA-29a in placenta-derived exosomes. These findings are somewhat consistent with previous study which found changes in miRNAs expression following an LPS engagement of TLR4 in different cell types. They demonstrated that these miRNAs regulate TLR-signaling pathways involved in inflammation through transcriptional regulation, including NF-κB and AP-1 (18). Of note, these findings are limited to the profile of vesicle-free miRNAs and did not identify exosomal microRNAs. However, limited studies have investigated the effect of LPS on the expression levels of exosomal microRNAs. One of these studies showed that LPS could change the exosomal miRNA expression patterns in cells such as macrophages, which is consistent with our data (19).

Although previous studies demonstrated that LPS could alter exosomal miRNA expression patterns in various cells, the effect of LPS on miRNAs expression in placenta-derived exosomes is still unclear. To the best of our knowledge, this study for the first time shows that LPS can change the exosomal miRNA expression pattern, especially the levels of miRNAs with immunomodulatory functions such as miRNA-29a in placenta cells. It seems possible that the production of inflammatory mediators and cytokines from immune cells at the fetal–maternal interface after exposure to LPS (14, 20) is related to the regulation of the expression of exosomal miRNA in placenta cells.

Our findings showed that the expression levels of miR-17 decrease in placenta-derived exosomes isolated from LPS-induced abortion mice, but this difference was not statistically significant. This data must be interpreted cautiously because the immunomodulatory effects of miR-17 on the regulation of inflammatory reactions are controversial. Philippe and coauthors showed that bacterial lipoprotein downregulates the expression of the miR-17 in rheumatoid fibroblast-like synoviocytes (21). They claimed that miR-17 can act as negative regulators of inflammation by downregulating IL-6 and matrix metalloproteinase 3. In another study, it was revealed that miR-17 can control Th1 responses through protecting cells from apoptosis, supporting IFN-γ production, suppressing inducible regulatory T-cell differentiation, etc. (22). Another study also showed that “miR-17 and miR-19b are the two miRNAs, responsible for promoting T${}_{H}$17 responses” (23). Because of the previous studies reporting a contrary function for miR-17 (21, 23), it is difficult to explain our result based on them. In the present study, we evaluated the expression levels of exosomal miR-17 in contrast to the earlier findings that evaluated vesicle-free miR-17. Considering the difference in stability and efficiency between vesicle-free miRNAs and exosomal microRNAs (24, 25), it is possible, therefore, that decreasing the expression of exosomal miR-17 in placenta cells after exposure to LPS upregulates genes involved in inflammatory responses.

Prior studies have noted the importance of miR-29a in pregnancy outcomes. In one of these studies, it was showed that villus miR-29a significantly upregulate in recurrent abortion women compared to normal pregnant women. They suggested that miR-29 might be involved in recurrent abortion pathogenesis (26). Considering the importance of miR-29 in pregnancy, we evaluated the levels of miR-29 in placenta-derived exosomes isolated from LPS-treated pregnant mice. Our findings showed that the levels of miRNA-29a significantly increase in them. This finding agrees with Tang and colleagues. They demonstrated that miR-29 regulate inflammatory responses to LPS in macrophages through the Akt1/NF-κB pathway. They also found the overexpression of miR-29 is associated with increased expression of proinflammatory cytokines (27). LPS may increase levels of proinflammatory cytokine expression at the fetal–maternal interface through the upregulation of exosomal miR-29 derived from placenta cells.

## 5. Conclusion

The results of the present study suggest that maternal exposure to LPS derived from the cell walls of gram-negative bacteria during pregnancy can alter miRNAs expression pattern into placenta-derived exosomes, especially miRNAs with immunomodulatory effects such as miR-29a and miR-17. However, we need to develop a full picture of the effect of LPS on the patterns of expression of exosome-derived miRNAs during pregnancy. Further studies are required to determine their expression levels in exosome isolated from decidual cells and plasma.

##  Conflict of Interest

The authors declare that they have no competing or conflicting interests.
